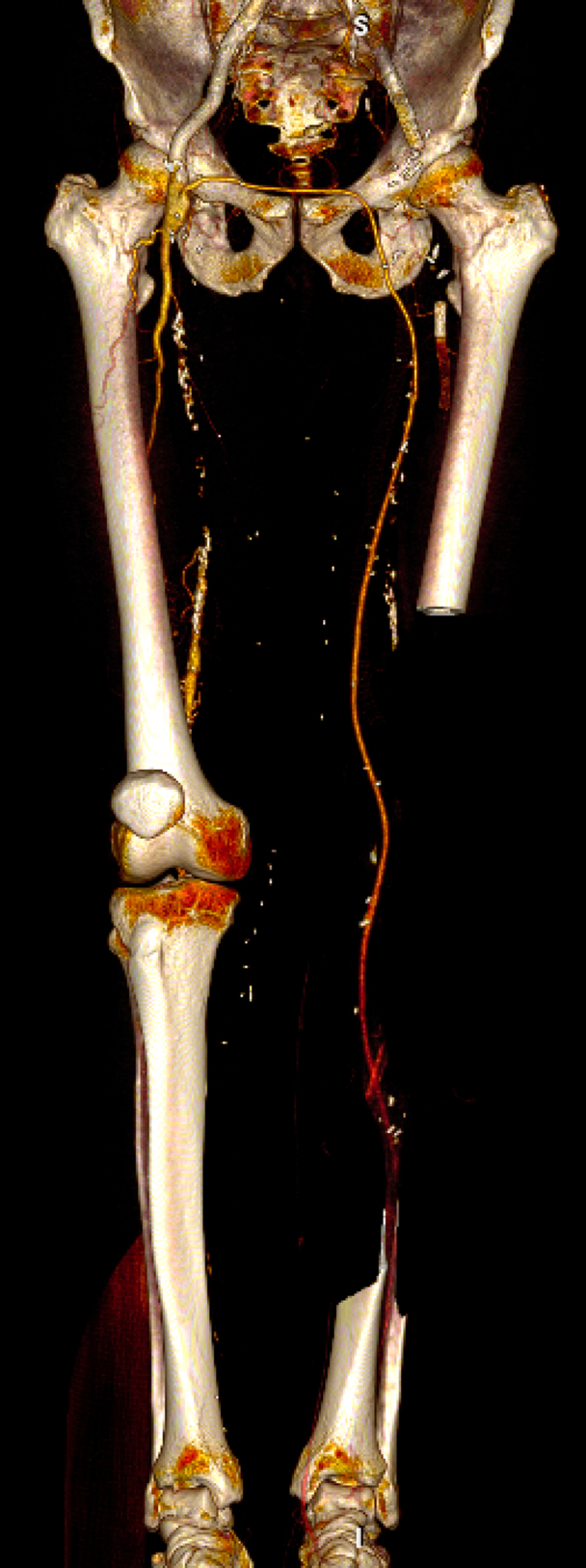# Continuous femoro-peroneal crossover bypass: A feasible technique for a last-chance revascularization

**DOI:** 10.1016/j.jvscit.2025.102009

**Published:** 2025-10-10

**Authors:** Federico Pascucci, Thomas Beaucarne, Sebastien Multon, Joseph Touma

**Affiliations:** a Department of Vascular Surgery, Henri Mondor University Hospital, Creteil, France; b Faculté de Santé, Université Paris Est Créteil, Creteil, France

A 74-year-old man with a past medical history of noninsulin-dependent diabetes mellitus, past smoking, chronic obstructive pulmonary disease, and hypertension presented to our attention with chronic limb-threatening ischemia of his left lower limb and a complex vascular history. In his past surgical history, he had already undergone a bilateral iliac artery stenting, a right common femoral artery endarterectomy, a left prosthetic ilio-femoral bypass, a left femoropopliteal bypass, a left deep femoral artery stenting, and a redo ilio-femoral bypass using ipsilateral great saphenous vein for a pseudoaneurysm. In the meantime, multiples episodes of acute left lower limb ischemia were treated with thrombectomy or thrombolysis. The patient came to our attention with chronic limb-threatening ischemia with rest pain, after his referral center proposed him a transfemoral amputation, considering any revascularization technique impossible. The preoperative computerized tomography angiography showed an occlusion of the left ilio-femoro-popliteal axis from the left common iliac artery to the peroneal artery (PA) (*A*). Any revascularization originating from the left femoral tripod was not considered feasible (*B*). A duplex ultrasound (DUS) confirmed the regular patency of the PA at its middle third. Considering the preoperative imaging and the severe left lower limb ischemia, we performed a continuous crossover bypass from the right common femoral artery to the left PA using the right reversed great saphenous vein. The vein portion between the two Scarpa’s triangles was tunneled in the Retzius space; the femoro-distal portion was tunneled beneath the Sartorius muscle. The patient did not report any intra- or postoperative complications. The total hospital stay was 13 days, and the patient was discharged home after immediate postoperative rehabilitation under aspirin 75 mg/day and rivaroxaban 20 mg/day. Patient was visited after 1 and 6 months with the results of a DUS. At the 12-month follow-up, the patient is completely asymptomatic, with no limiting claudication or rest pain of the lest leg nor steal phenomenon on the right side, with a regular bypass primary patency confirmed by a computerized tomography angiography and a DUS. Ankle-brachial index is 0.93 on the left side and 0.86 on the right side (*C*/Cover).

To the best of our knowledge, continuous femoro-peroneal crossover bypass has not been documented yet in the literature; however, femoro-popliteal crossover bypass cases have already been reported.^1^^,^^2^

In our opinion, this case highlights how extreme surgical revascularizations, even in highly complex scenarios, can avoid major amputation and have very satisfying functional results.

**Figure:**
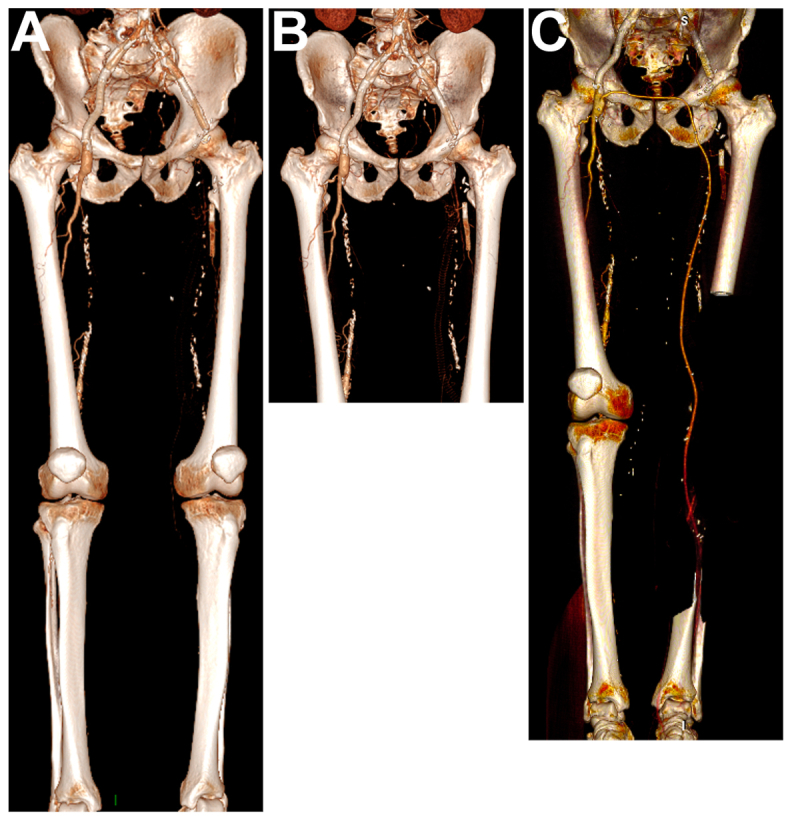

## Funding

None.

## Disclosures

None.

## Figures and Tables

**Cover image:**